# Evolution of S-domain receptor-like kinases in land plants and origination of S-locus receptor kinases in Brassicaceae

**DOI:** 10.1186/1471-2148-13-69

**Published:** 2013-03-19

**Authors:** Shilai Xing, Mengya Li, Pei Liu

**Affiliations:** 1 Department of Ecology, College of Resources and Environmental Sciences, China Agricultural University, Beijing 100193, People’s Republic of China

**Keywords:** SRLK, SRK, SRLCK, Gene fusion/fission, Neo-subfunctionalization

## Abstract

**Background:**

The S-domain serine/threonine receptor-like kinases (SRLKs) comprise one of the largest and most rapidly expanding subfamilies in the plant receptor-like/Pelle kinase (RLKs) family. The founding member of this subfamily, the S-locus receptor kinase (SRK), functions as the female determinant of specificity in the self-incompatibility (SI) responses of crucifers. Two classes of proteins resembling the extracellular S domain (designated S-domain receptor-like proteins, SRLPs) or the intracellular kinase domain (designated S-domain receptor-like cytoplasmic kinases, SRLCKs) of SRK are also ubiquitous in land plants, indicating that the SRLKs are composite molecules that originated by domain fusion of the two component proteins. Here, we explored the origin and diversification of SRLKs by phylogenomic methods.

**Results:**

Based on the distribution patterns of SRLKs and SRLCKs in a reconciled species-domain tree, a maximum parsimony model was then established for simultaneously inferring and dating gene duplication/loss and fusion /fission events in SRLK evolution. Various SRK alleles from crucifer species were then included in our phylogenetic analyses to infer the origination of SRKs by identifying the proper outgroups.

**Conclusions:**

Two gene fusion events were inferred and the major gene fusion event occurred in the common ancestor of land plants generated almost all of extant SRLKs. The functional diversification of duplicated SRLKs was illustrated by molecular evolution analyses of SRKs. Our findings support that SRKs originated as two ancient haplotypes derived from a pair of tandem duplicate genes through random regulatory neo-/sub- functionalization in the common ancestor of the Brassicaceae.

## Background

The “S domain” (SD) was initially defined by the S-locus glycoprotein (SLG) and the S-locus receptor kinase (SRK), which are encoded by two closely-linked genes in the Brassica self-incompatibility (SI) determining locus, the *S* locus. *SLG,* which was the first *S* locus-derived gene identified, encodes a secreted glycoprotein, whereas *SRK* encodes a transmembrane receptor kinase with an extracellular domain that shares extensive sequence similarity with SLG [1]. SRK is the female determinant of specificity in “self-pollen” recognition, and in self-incompatible species of the Brassicaceae (crucifers), the *S* haplotype-specific binding of SRK to its cognate pollen-borne ligand *S* locus cystein rich protein/*S* locus protein 11 (SCR/SP11) activates the SRK and triggers a signaling cascade that culminates in “self-pollen” rejection [2,3]. The extracellular S domain of SRK is responsible for ligand binding [4,5], whereas the intracellular kinase domain (KD) is thought to translate this signal into a cellular response by phosphorylating Arm Repeat Containing (ARC1) protein, an E3 ligase involved in protein ubiquitination [6,7]. With the increasing availability of sequenced plant genomes, it has been realized that proteins having a structure resembling SRKs, designated S-domain receptor-like kinases (hereafter SRLKs), form one of the largest and most-rapidly expanding subfamilies within the plant receptor-like/Pelle kinase superfamily [8-11]. In addition, a large group of receptor-like cytoplasmic kinases (RLCKs) resembling the intracellular kinase domains of SRLKs but lacking the extracellular S-domain (designated S-domain receptor-like cytoplasmic kinase, SRLCKs) were also defined by their close phylogenetic relationship to the kinase domains of SRLKs [8,12]. Interestingly, another class of proteins resembling SLGs, designated S-domain receptor-like proteins, SRLPs, is also ubiquitous in plants [13-15], suggesting that the composite SRLKs likely originated by fusion of the two split component proteins. Phylogenetic analyses of the kinase domains in *Arabidopsis thaliana* also suggested that SRLKs are not monophyletic and probably arose *via* independent recruitment of S domains [10].

Gene fusion is considered to be an important evolutionary path to create novelty in protein architectures (the linear arrangement of protein domains) and functions by forming composite proteins and linking components of extant signaling/biochemical pathways [16]. In agreement with this notion, a number of chimeric genes generated by gene fusions have been reported to have important functions [17-20]. Based on the assumption that selection favors fusions of functionally-related proteins, identification of fusion-link (split proteins in some genomes and fused proteins in other genomes) has initially been used to predict genome-wide protein interactions and functions in the extremely compact genomes of prokaryotes and yeasts [16,21], and more recently in the more complex eukaryotic genomes [22,23]. Based on the distribution patterns of the composite proteins and the split proteins in either the species trees [23] or the domain trees [24], gene fusion events were inferred in a large number of sequenced genomes. Furthermore, a maximum parsimony algorithm has been established to analyze the evolution of protein architectures, in particular domain fusion and fission, based on the inferred ancestral architecture at each node in the species trees [25] or domain trees [25,26]. In plants, because only the Arabidopsis and rice genomes have been included in such studies, very little is known about the evolution of domain architecture in other plant genomes. Despite the fact that multiple-domain proteins in super-protein families are normally composed of abundant and versatile domains and tend to undergo more independent gene fusion/fission events [27], analysis of gene fusion/fission events in a large gene subfamily such as SRLK subfamily is still lacking.

As the only members of the SRLK subfamily whose function is known, SRKs are well suited to investigate the functional diversification of SRLKs. In Brassica species but not Arabidopsis species, SRKs may be clearly divided into two classes, class I and class II [28]. Moreover, phylogenetic analyses of SRK kinase domains showed that SRKs from Brassica species are not monophyletic, having descended from only two of the lineages that were presumably present in the Arabidopsis-Brassica ancestor, and that diversification of the Brassica *S* haplotypes took place after the separation of the two genera [29]. Furthermore, theoretical analyses have long predicted that SI could have been first expressed in a two *S-*haplotype system causing incomplete suppression of selfing, with further differentiation among *S* haplotypes and enhancement of SI expression having evolved subsequently [30,31]. This long-held hypothesis awaits further elaboration by molecular evolution studies.

In this study, we first retrieved SRLKs from five sequenced genomes representing the major lineages of land plants. SRLCKs were then delineated by their close phylogenetic relationship to SRLKs based on a maximum likelihood (ML) kinase domain tree. On the basis of a reconciled species-domain tree including both SRLKs and SRLCKs, gene duplication/loss and fusion events in SRLK evolution were inferred and dated by integrating the maximum parsimony ancestral architecture inference algorithm [25,26] into the widely applied gene duplication/loss model [32]. In addition, the origination of SRKs in the Brassicaceae was explored by reconstruction of SRK kinase domain phylogeny in the context of SRLK evolution.

## Results

### SRLKs emerged in early land plants and expanded greatly in Angiosperms

**Figure 1:**
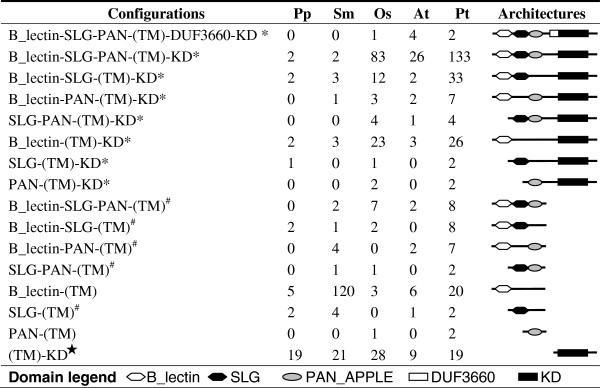
**Number and domain architectures of SRLKs, SRLPs and SRLCKs from the five species included.** Domain configurations of SRLKs (indicated by ^*^), SRLPs (indicates by ^#^) and SRLCKs (indicated by ★ ) from moss (Pp), spikemoss (Sm), *Arabidopsis* (At), rice (Os) and poplar (Pt) are included in our dataset. (TM) indicates that a TM domain may or may not be predicted.

SRLKs are composed principally of three modular domains in a configuration of S domain (SD)-transmembrane domain (TM)-kinase domain (KD). SDs are further divided into three subdomains in a configuration of B_lectin-SLG-PAN_APPLE (Figure 1). The B_lectin and PAN_APPLE domains have been proposed to be important for protein structure and dimerization of Brassica SRKs, while the hypervarible SLG domain plays a key role in SCR binding [4,5]. Transmembrane domain (TM) prediction is not always accurate, thus TM will not be considered in our protein architecture analyses. Proteins with stand-alone SLG and PAN_APPLE domains are rare, whereas B_lectin domain proteins are more abundant, particularly in the spikemoss genome (Figure 1).

A total of 253 SRLK and 19 SRLP sequences with the domain architecture typical of SRKs (B_lectin-SLG-PAN_APPLE-TM-KD) or SLGs (B_lectin-SLG-PAN_APPLE) were retrieved and are hereafter referred to as typical SRLKs and SRLPs respectively (Figure 1 and Additional file 1: Table S1). Other combinations of B_lectin, SLG, PAN_APPLE, and KD domains are also ubiquitous in different plant species (Figure 1). These combinations may represent either the precursors of typical SRLKs/SRLPs or the degenerated products of the typical SRLKs/SRLPs. Our search also retrieved stand-alone B_Lectin and PAN_APPLE sequences in the genomes of green algae *Chlamydomonas reinhardtii* and *Ostreococcus tauri*, whereas sequences of stand-alone SLG domains or any combination of B_lectin, PAN_APPLE, SLG, and KD domains were not detected. In view of the fact that plant RLKs were likely generated after the divergence of land plants from green algae [8], we tentatively included sequences containing combinations of at least two of the four modular domains, adding 139 SRLK and 30 SRLP sequences with atypical domain architectures to our dataset. Nine proteins with stand-alone SLG, which characterize S-domains, were also included as SRLPs. In total, our dataset included 7, 9, 129, 38 and 209 SRLKs and 4, 12, 10, 5 and 27 SRLPs from the genomes of *Physcomitrella patens* (moss), *Selaginella moellendorffii* (spikemoss), *Oryza sativa* (rice), *Arabidopsis thaliana* (Arabidopsis), and *Populus trichocarpa* (poplar) respectively (Additional file 1: Table S1). In moss and spikemoss, a relatively small number SRLKs (7 and 9, respectively) are found, and SRLKs continued to expand immediately after the divergence of angiosperms, since there are 23.8, 19.3 and 33.9 times (normalized by genome size) as many members in rice, Arabidopsis and poplar, respectively, compared with moss.

### Kinase domain tree and delineation of SRLCKs

**Figure 2:**
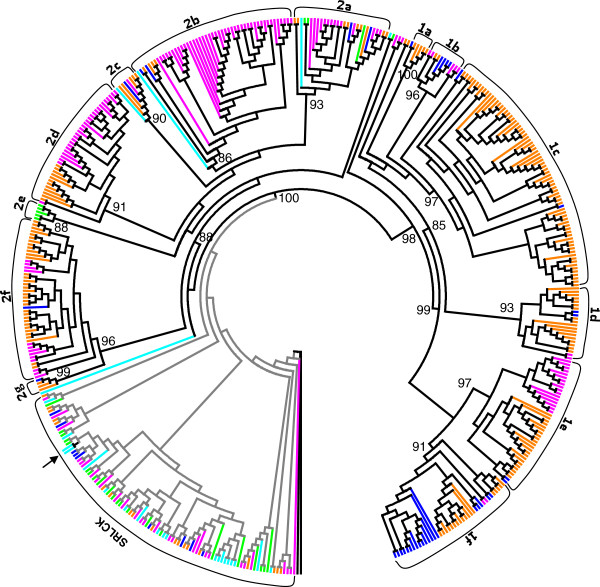
**Distribution patterns of SRLKs and SRLCKs and classification of SRLKs.** The ML phylogenetic tree (Additional file 1: Figure S1) was displayed in circular form to show the classification of SRLKs (subgroups 1a-1f and 2a-2g are evident in each of the SD-1 and SD-2 groups) and the distribution patterns of SRLKs and SRLCKs from five land plants (electric blue for moss; bright green for spikemoss; deep pink for rice, orange for poplar, and blue for Arabidopsis). The roots are shown by branches in filled black lines and the aLRT bootstrap values of the major internal nodes are indicated by numbers. Predicted two gene fusion events are labeled by branches in black, the minor fusion event is indicated by black arrow.

To delineate SRLCKs by the kinase-domain tree, KD sequences from 392 SRLKs in our dataset and total 96 RLCKs of the five species from Phytozome V9.0 that were the best hits to the members of the gene family HOM000017 in PLAZA 2.5 database were then used to construct a maximum likelihood (ML) phylogenetic tree (Figure 2 and Additional file 2: Figure S1). The basal nodes of the phylogenetic tree are composed of 17 RLCKs, while two well-supported monophylectic groups (with a 100 bootstrap) in land plants are evident. The first group consists of 390 SRLKs, while the second group consists all 79 RLCKs as well as 2 moss SRLKs. Therefore, the 79 RLCKs are hereafter designated as SRLCKs (Additional file 1: Table S1 and Additional file 2: Figure S1). Considering protein architectures, SRLKs are not monophylectic but form two groups, the major group containing 390 SRLKs and the minor group containing 2 moss SRLKs. In the major group, two large subgroups correspond to the previously-identified SD-1 and SD-2 S-domain RLKs [8,9], which are composed of 202 SRLKs, and 188 SRLKs, respectively.

SD-1 SRLKs are specific for angiosperms, indicating that this group is approximately 140 million years old [33]. In contrast to SD-1, SD-2 is a very diverse group of SRLKs from all species with well-dissolved clades. Given that the oldest evidence for the existence of vascular plants is found in Upper Ordovician, SD-2 SRLKs are inferred to be more than 450 million years old (Figure 2 and Additional file 2: Figure S1). Based principally on the topology of the trees, clade support values and branch length, we tentatively defined 6 (SD-1a, SD-1b, SD-1c, SD-1d, SD-1e, SD-1f) and 7(SD-2a, SD-2b, SD-2c, SD-2d, SD-2e, SD-2f and SD-2g) subgroups in each of the SD-1 and SD-2 groups. Interestingly, the *A. thaliana* SRK falls in the SD-1b subgroup, sharing a most recent common ancestor with 3 Arabidopsis, 5 rice, and 2 poplar SRLKs (Figure 2 and Additional file 2: Figure S1). More importantly, except for the two moss SRLKs, all SRLKs or SRLCKs cluster together, which strongly suggests that extant SRLKs are likely derived from one major S-domain recruitment event in land plant evolution.

### Inference and dating of gene duplication/loss and fusion/fission events in the evolutionary history of SRLKs

**Figure 3:**
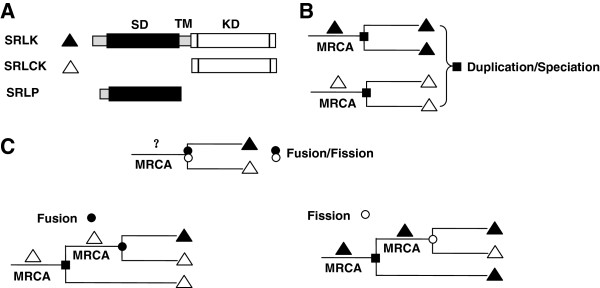
**Illustration of a model for inferring the protein architecture of most recent common ancestors (MRCAs).** Based on the distribution patterns of SRLKs and SRLCKs in a kinase-domain tree, gene duplication/speciation and gene fusion/fission events were inferred. (**A**) Protein architecture of SRLKs (represented by filled triangles), SRLCKs (represented by open triangles), and SRLPs. (**B**) When the two leaves on bifurcating branches are of the same architecture (either SRLK or SRLCK), a duplication or speciation event is inferred. (**C**) When the two leaves are of different architectures (one SRLK and one SRLCK), the ancestral architectures at the inner nodes were inferred by the leaf architectures of the deeper branch as illustrated.

**Figure 4:**
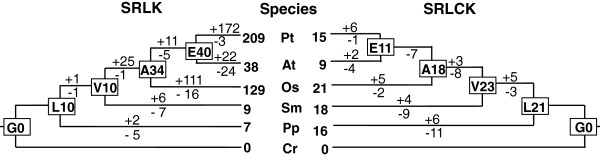
**Gene gain and loss of SRLKs and SRLCKs in the evolution of land plants.** The names of internal nodes and species are abbreviated (L, Land plants; V, Vascular plants; A, Angiosperms, E, eudicots; Pt, *Populus trichocarpa*; At, *Arabidopsis thaliana*; Os, *Oryza sativa*; Sm, *Selaginella moellendorffii*; Pp, *Phycomitrella patens*). The numbers of common ancestors at the four internal nodes (L, V, A and E) are shown in the rectangles. Numbers after the plus signs indicate the numbers of gene gain events, whereas numbers after the minus signs indicate gene loss events.

The gene duplication-loss model embedded in Notung has been used to infer and date gene duplication/loss events in RLK evolution [8]. After a reconciled species-domain tree was generated by Notung, we inferred the ancestral architectures of all nodes along the reconciled tree according to our model (Figure 3 and Additional file 3: Figure S2). Gene duplication/loss and fusion/fission events were inferred and dated. SRLKs were shown to have a rapid gene expansion, whereas SRLCKs tended to have undergone a decay comparing with the inferred number of ancestors in early land plants (Figure 4). A leap of common ancestor number from 10 in the ancestor of land plants to 34 of angiosperms represents a critical stage in SRLK expansion, which coincides with the emergence of angiosperms and the rapid increase in gene duplication events. In the past 450 million years after the divergence of moss and the embryophyta ancestor, two duplications of SRLKs was inferred, while as many as 172 duplications were inferred to have occurred in the past 100 million years after the divergence of poplar and its rosid ancestor. In Arabidopsis, the results suggest rapid birth and death of SRLKs, and the loss of most SRLCKs (Figure 4).

Based on the distribution patterns of SRLKs and SRLCKs in the reconciled tree, gene fusion/fission events in the evolutionary history of SRLKs were also inferred (Figure 2 and Additional file 3: Figure S2). In total, 2 gene fusion events were inferred, while no fission event was detected. The major ancient gene fusion event occurred in the common ancestor of land plants and generated the ancestral gene of 390 extant SRLKs. The other minor gene fusion event occurred after moss have diverged from the common ancestor of land plants, generating two extant moss SRLKs. Notably, scarcity of gene fusion and the lack of fission events in the evolution of SRLKs subfamily suggests that functional diversification of SRLKs is principally driven by sub-or neo-funtionalization of duplicated genes.

### Origination of brassicaceae SRKs in the context of SRLK evolution

**Figure 5:**
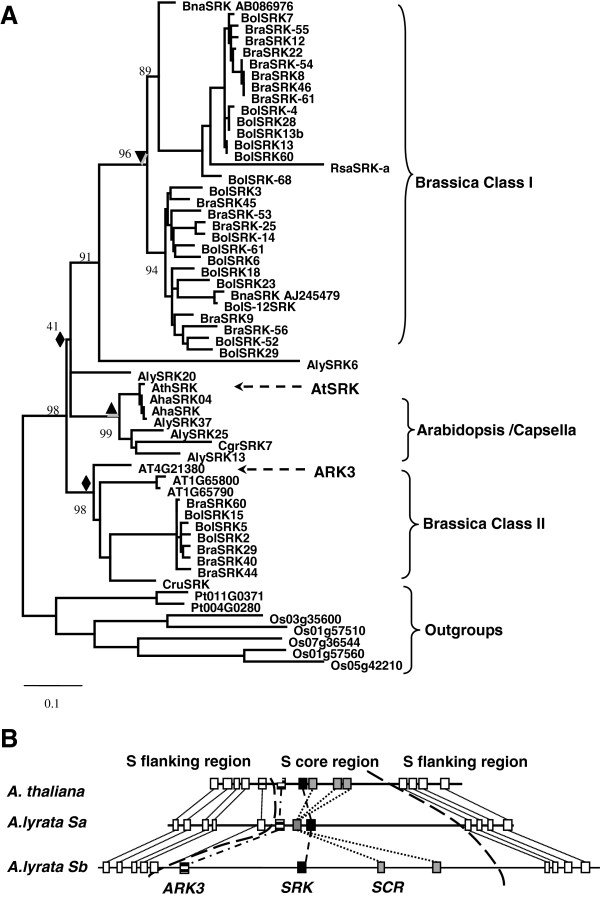
**Phylogeny and evolution of SRKs in the Brassicaceae. **(**A**) A ML phylogenetic tree of KDs was constructed by including all members of the SD-1b group and 47 full-length SRKs from Brassicaceae species. The aLRT bootstrap (in bold) values of the major internal nodes are indicated by numbers. An orthologous group in the Brassica/Raphanus lineage (indicated by an inverted filled triangle), an orthologous group in the Arabidopsis/Capsella lineage (indicated by a filled triangle), and two orthologous groups in the Brassicaceae (indicated by filled diamonds) are shown. The diamond between brackets indicates the orthologous group with relatively low support value. The names of Brassicaceae species are abbreviated (Ath, *Arabidopsis thaliana*; Aly, *Arabidopsis lyrata*; Aha, *Arabidopsis halleri*; Cru, *Capsella rubella*; Cgr, *Capsella grandiflora*; Bol, *Brassica oleracea*; Bra, *Brassica rapa*; Bna, *Brassica napus*; and Rsa, *Raphanus sativus*). (**B**) Structural comparison of the *A. thaliana* and *A. lyrata* haplotypes *Sa* and *Sb* (modified from [36]).

The *A. thaliana* SRK falls within the SD-1b group, a small monophyletic group in angiosperms that includes 10 non-SRK members, which are good candidates to investigate the functional diversification of SRLKs (Figure 2). We thus performed phylogenetic analysis of SRKs by including KD sequences of 47 SRKs from the Brassica/Raphanus and Arabidopsis/Capsella lineages as well as SD-1b members (Additional file 4: Table S2 and Figure 5A). A functional SRK from self-fertile *A. thaliana* accession Wei was included to replace the ΨSRK (At4g21370), because its function in SI has been demonstrated [34]. In addition, a likely functional SRK sequence (CruSRK) from the newly-evolved self-fertile species *Capsella rubella* is also included [35].

The topology of our rooted ML kinase domain tree is similar to that of an unrooted ML kinase domain tree based on nucleotide sequences [29]. Three separate well-supported clades, Brassica class I, Arabidopsis/Capsella, and Brassica class II, are evident (Figure 5A). The *A. thalina* non-SRK SRLKs, Arabidopsis Receptor Kinase (ARK1/ARK2/ARK3), do not fall within the outgroups in the kinase domain tree (Figure 5A), but form a well-supported orthologous group in the Brassicaceae with Brassica class-II SRKs. In contrast, they do fall within outgroups in S-domain trees in our (Additional file 5: Figure S3) and other studies [37-39]. *A. thaliana* SRK (At4g21370) and most SRKs from *A. lyrata*, *A. halleri,* and *Capsella grandiflora* also form a distinct orthologous group in the Arabidopsis/Capsella lineage. In addition, Brassica class-I SRKs and Arabidopsis/Capsella SRKs (except for AlSRK20) appear to form a large orthologous group in Brassicaceae, albeit with a relatively low bootstrap value (Figure 5A). *A. thaliana SRK* (*At4g21370*) and *ARK3* (*At4g21380*) are located in different orthologous groups and are arranged in tandem (Figure 5B). The same arrangement of *SRK* and *ARK3* orthologs was also retained in all characterized *S* haplotypes of *A. lyrata* (Figure 5B).

## Discussion and conclusions

The architecture of the SRLKs was likely established after the divergence of land plants from green algae approximately 1000 million years ago, but before the divergence of vascular plant lineage from the moss lineage. Consistent with their predicted function in perceiving various external signals, the SDs of SRLKs are very variable in both sequence and architecture. Because of the highly variable nature of the SDs and the resulting poor sequence alignments, it was difficult to use these domains for investigating the trajectory of SRLK evolution. In contrast, kinase-domain sequences are more conservative likely due to constrains imposed by the requisite interactions with other signaling partners, and were thus used in our study to simplify the interpretation of SRLK evolution.

By integrating the ancestral architecture inference algorism [26] into the widely applied gene duplication-loss parsimony model [8,32], we established a maximum parsimony model suitable to infer and date gene duplication/loss and fusion/fission events in *SRLK* evolution (Figure 3). Our results suggest that almost all (except for 2 moss SRLKs) SRLKs of land plants are derived from a single ancient domain fusion event. Continuous expansion of SRLKs by gene duplication has played pivotal roles in shaping the phylogenies of extant SRLKs. In contrast with previous interpretations based merely on topology of the phylogenetic trees [8-10], we show that SD-1 and SD-2 group SRLKs were generated by the same ancient gene-fusion event that likely occurred in the common ancestor of land plants. Mis-annotation of genomic sequences, however, may be accounted for the inconsistence between our results and the published papers [8-10]. When the poplar genome of Phytozome v6.0, in which only 10% annotated gene models are supported by full length cDNAs, were used for detecting fusion/fission events, we could detect 5 fusion events and 23 fission events occurred in poplar. However, no fusion or fission event was found using the updated poplar genome of Phytozome v9.0, in which 218 out of 228 SRLKs/RLCKs were supported by assembly ESTs (Additional file 1: Table S1). As in all other such studies, we assigned an equal cost for gene fusion, fission, duplication, loss, or speciation in order to avoid prior bias stemming from uncertainties relating to the relative frequency of these events [26] and their dependence on the particular genomes investigated [22,23]. Since the distribution patterns of the composite SRLKs and the split SRLCKs on the reconciled tree are critical for our analysis, expansion and high-rate retention of both the composite *SRLK* and split *SRLCK* genes is essential. We might have underestimated the actual number of gene fusion/fission events in our analyses because the domain architectures (composite or split) of the most recent common ancestors (MRCAs) at the leaf nodes with lost genes can only be inferred by the parsimony principle (Additional file 3: Figure S2). Similar requirements can be largely fulfilled in most RLK subfamilies such as Lysin motif-type RLKs [40]. We thus propose that our method could be extrapolated to analyze gene fusion/fission events in other multiple-domain super-protein families.

**Figure 6:**
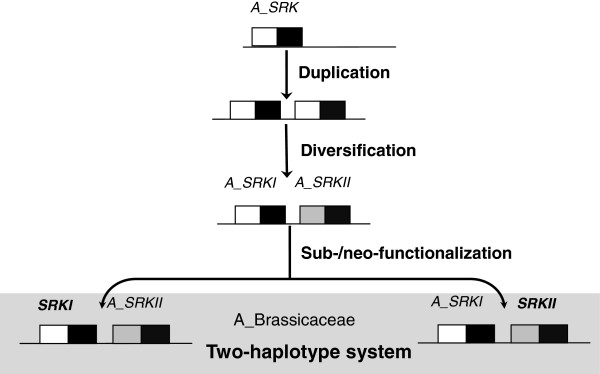
**Proposed model for the evolution of the ancient two-haplotype SRK system.** The duplication and diversification of the common ancestor of Brassicaceae SRKs, *A_SRK*, generated two tandem duplicate genes *A_SRKI* and *A_SRKII*. Random sub-/neo-functionalization of *A_SRKI* and *A_SRKII* produced the postulated two-haplotype system of two ancient SRK haplotypes *SRKI* and *SRKII*.

**Figure 7:**
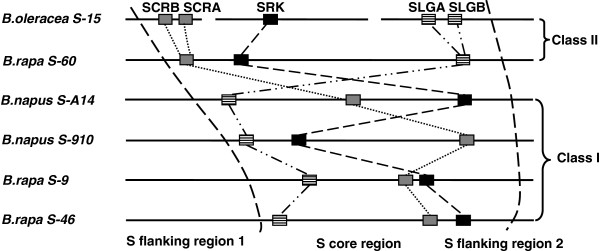
**Different gene order of SRKs relative to SLGs in Brassica class-I and class-II S haplotypes.** The gene order of SRKs and SLGs from two class-II *S* haplotype (*B. rapa* S-60 and *B. oleracea S-15*) and 4 class-I haplotypes (*B. rapa* S-9 and S-46; *Bnn napus* S-A14 and S-910) is compared. The arrangement of the *S* loci was determined by molecular markers in the *S*-locus flanking region 1 and region 2 (modified from [54]).

**Figure 8:**
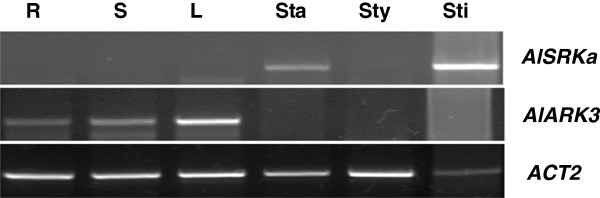
**Complementary expression domains of AlSRKa and AlARK3 in A. lyrata.** Expression of *AlARK3* and *AlSRKa* were analyzed in root (R), stem (S), leaf (L), stamen (Sta), style (Sty), and stigma (Sti) tissues by RT-PCR using gene-specific primers. *ACT2* was included as the loading control.

Functional diversification following the origination of multiple-domain proteins is a common theme in studies of protein family evolution [41-43]. Although great efforts have been devoted to investigate the diversification of SRKs by reconstructing their phylogeny, such studies are limited by the lack of suitable outgroups [29]. Utilizing SD-1b members as outgroups, we show that in the Brassicaeae, SRKs do not form a monophyletic clade. More intriguingly, ARK1/ARK2/ARK3 are confidently clustered with Brassica class-II SRKs, forming a distinct orthologous group in the Brassicaceae (Figure 5B). This orthologous relationship between ARK1/ARK2/ARK3 and class-II SRKs has not been previously revealed because S-domain trees were used in most studies [28,38,39]. Even in studies that used the kinase-domain trees, neither ARK1/ARK2/ARK3 [29] nor Brassica SRKs were included [37,38]. Furthermore, *A. thaliana* SRK confidently clustered with most SRKs of Arabidopsis/Capsella species, forming another distinct orthologous group in the Arabidposis/Capsella lineage (Figure 5B). Since *SRK* and *ARK3* are arranged in tandem in the *S* haplotypes of Arabidopsis species, we propose the following model on the origins of SRKs. An ancient duplication in the common ancestor of Brassicaceae produced two tandemly-arranged paralogous genes, designated as ancestor of *SRKI* (*A_SRKI*) and ancestor of *SRKII* (*A_SRKII*). Subsequently, random neo-fuctionalzation and/or sub-funtionalization of *A_SRKI* and *A_SRKII* produced two ancient *S* haplotypes, from which *SRKs* might have been derived by further diversification (Figure 6). This model provides a mechanism for the establishment of the long-proposed ancient two-*S* haplotype SI system in the common ancestor of the Brassicaceae [30,31]. However, the clustering of Arabidopsis/Capsella SRKs with Brassica/Raphanus class-I SRKs in the same orthologous group remains ambiguous because of the relatively low support value. The different gene orders of *SLGs* and *SRKs* in known class-I and class-II *S* haplotypes (Figure 7) and the inference that SLGs were derived from an ancient duplicated copy of SRK [1,36,44], support the notion that class-I and class-II SRKs might also be derived from two tandemly duplicated paralogous genes. Similar examples of the evolution of functional orthologous genes from ancient paralogs have been reported for *AGAMOUS* and *PLENA*[45] as well as *SRnases* in diploid strawberry [46]. Paralogs produced by tandem duplications such as *A_SRKI* and *A_SRKII* might be prone to this random evolution, because they are free from constrains of different genomic contexts. Regulatory neo-functionalization is the most likely evolutionary scenario for paralogs produced by tandem duplications [47]. However, no overlap in the expression domains of *ARK3* and *SRKa* in self-incompatible *A. lyrata* has been observed in either vegetative or floral tissues (Figure 8), suggesting that regulatory sub-functionalization played a significant role in SRK evolution. Nevertheless, certain SRK variants are expressed in leaf tissue [48], indicating that regulatory neo-functionalization also played a role. Together with the fact that *Arabidopsis* ARK3 is orthologous to Brassica class-II SRKs, our findings indicate that the biochemical functions of SRKs and ARK3, and probably other SRLKs in the SD-1b group, are likely the same or very similar. This conclusion is in line with the existence of a conserved DUF 3660 motif between SDs and KDs in ARK1, ARK2, ARK3, and all SRKs, and particularly with the findings that ARK1, ARK2, and ARK3 interact with and phosphorylate a number of the Arabidopsis plant U-box/ARM-repeat (AtPUB-ARM), ARC1 homologs of *A. thaliana*[49]. In view of that ARKs and other SRLKs may be involved in plant innate immunity responses [50-53], the overlap of SRK signaling with that of plant immunity mediated by ARK3 and other SD-1b group SRLKs suggested by our molecular evolution study is intriguing and should be pursued by further experimental investigations.

## Methods

### Sequence retrieval

*Physcomitrella patens* (moss), *Selaginella moellendorffii* (spikemoss), *Oryza sativa* (rice), *Arabidopsis thaliana* (Arabidopsis), and *Populus trichocarpa* (poplar), which represent the major lineages in land plant evolution, were used in this study. The annotated protein sequences of the 5 sequenced genomes were downloaded (http://www.phytozome.net). To identify SRLK and SRLP sequences, an HMMer search was performed by the standard profiles of the modular domains of S-domains [5], the B_lectin, SLG, and PAN_APPLE domains. After searching the sequences of primary screening against the Pfam database with the established “trusted cut off”, we detected in the moss, spikemoss, rice, Arabidopsis, and poplar genomes, respectively, 13, 136, 134, 47, and 244 B_lectin containing proteins; 9, 13, 111, 36, and 194 SLG-containing proteins; as well as 2, 10, 102, 37 and 167 PAN_APPLE-containing proteins (Additional file 1: Table S1). Because proteins belonging to the same homologous group are readily and confidently retrieved from PLAZA 2.0 database (http://bioinformatics.psb.ugent.be/plaza/), we thus retrieved RLCK sequences from the homologous group (HOM000017, which contain all SRLKs identified from Phytozome 9.0 ), and then updated each RLCK sequence with the best hit from Phytozome v9.0 using BLASTP. At last, these hits filtered for only one kinase domain using Pfam, were used as candidates for SRLCKs (Additional file 1: Table S1).

### Sequence alignment and phylogenetic analysis

The composite SRLKs and their split component proteins, SRLCKs and SRLPs (Figure 3A), are ubiquitous in plants suggesting the occurence of fusion and/or fission events. To explore the trajectory of SRLK evolution, a kinase domain tree including both SRLKs and SRLCKs was constructed. The amino-acid sequences of the kinase domains of 392 SRLKs and 96 RLCKs were aligned using ClustalX (Version 2.0) with Gonnet 250 protein weight matrix and the pairwise parameters of gap opening 10 and gap extension 0.2 [55] (Additional file 6: Sporting dataset 1) Arabidopsis homologs of Right Open Reading 1 (RIO1) family kinase (At 5 g37350 and At 2 g24990) were used as outgroups [11]. ProtTest v2.4 [56] was used to select the best-fit model of protein evolution for the alignment. Then, according to the best-fit model predicted by ProtTest v2.4, a rooted maximum likelihood (ML) tree was constructed with the JTT substitution model using the PhyML v3.0 online program, and the support of interior branches was assessed with the aLRT bootstrap method [57]. Finally, the phylogenetic tree was displayed and edited using MEGA v5.0 [58].

According to this phylogenetic tree, the *A. thaliana* SRK (At4g21370), ARK1 (At1g65790), ARK2 (At1g65800), and ARK3 (At4g21380) sequences as well as 7 SRLKs from rice and poplar form a well-supported subgroup. Kinase domain sequences from these 11 proteins and Brassicaceae 47 SRK sequences (Additional file 4: Table S2 and Additional file 7: Supporting dataset 2) were retrieved from Uniprot and were included to construct another ML tree using PhyML v3.0 online program. We included SRKs from as many Brassicacea species as possible, including *Brassica oleracea*, *B. rapa*, *B. napus,* and *Raphanus sativus* in the Brassica/Raphanus lineage and *A. thaliana*, *A. lyrata*, *A. halleri*, *Capsella grandiflora,* and *C. rubella* in the Arabidopsis/Capsella lineage.

### Inference and dating of gene duplication/loss and fusion/fission events

A reconciled species-domain tree was generated using the Notung program, which offers a unified framework for incorporating duplication-loss parsimony into phylogenetic analysis [32]. After reconciling the ML domain tree with the species tree of the five land plant species constructed using the NCBI Taxonomy Browser (http://www.ncbi.nlm.nih.gov/Taxonomy/CommonTree/wwwcmt.cgi), gene duplicate- on/loss and fusion events were inferred and dated. Based on the reconciled tree, the ancestral protein architectures were inferred by a modified maximum parsimonious protein ancestral architecture inference algorithm [25,26]. The extant protein architectures at the leaves are used to initialize the tree. Instead of traversing the whole tree, we infer the ancestral protein architecture of MRCA at each node sequentially from the leaves to the root. To avoid prior bias, gene duplication/loss, gene fusion/fission, and speciation were assigned an equal cost of 1. However, when a gene fusion/fission event occurred at a node, a gene duplication/speciation event must also have occurred, thus the node is assigned a cost of 2. In the bifurcating terminal branches of the reconciled species-domain tree, three configurations could be detected (Figure 3B and 3C). When both leaf nodes are of the same domain architecture (SRLK or SRLCK), we inferred that the internal node has a MRCA of the same architecture. The two proteins could be paralogs derived from a duplication event or orthologs generated by speciation events (Figure 3B). When the two leaf nodes are of different domain architectures (one SRLK and one SRLCK), the architectures of the MRCAs were inferred by those of the deeper branches. The trees were traversed twice and the ancestral architectures yielding the lowest cost were selected (Figure 3C). After the architectures of these outer nodes were inferred, they were treated as leaves to initiate another round of ancestral architecture-inferring process until the ancestral architectures at all inner nodes were inferred, after which gene duplication/loss and fusion/loss events were inferred and dated.

### RNA extraction and reverse transcription polymerase chain reaction (RT-PCR)

RT-PCR was used to examine the spatial expression of *AlSRK* and *AlARKs* in *A. lyrata* organs. Total RNA from root, stem, leaf, stamen, style, and stigma tissues of *A. lyrata* was isolated using the Trizol reagent (Invitrogen, USA) according to the manufacturer’s instructions. The residual genomic DNA in the total RNA was removed by treatment with RNase-free DNaseI and the total RNA was further purified with phenol chloroform-isoamyl alcohol. RT-PCR was performed using SuperScript™II RNase HI Reverse Transcriptase (Invitrogen, USA) using the following primer pairs: 5^′^-GACAACGCGTGTGAGACCTAT-3^′^ and 5^′^-CATTAGG AGCTGCAGTTGCTC-3^′^ for *AlSRKa,* and 5^′^-GACCAATGCGATGATTACAAAG -3^′^ and 5^′^-CAGTAGCTGTTTCAATTAGT-3^′^ for *AlARK3*. The PCR conditions of *AlSRKa*/*AlARK3* were 94°C for 3 min followed by 40 cycles of the following: 94°C for 30 s, 58°C/52°C for 30 s, and 72°C for 40 s. The amplification products were then analyzed with agarose gel electrophoresis.

### Availability of supporting data

The data sets supporting the results of this article are included within the article (and its additional files)

## Abbreviations

SRLK: S-domain serine/threonine receptor-like kinase; RLK: plant receptor-like/Pelle kinase; SRK: *S*-locus receptor kinase; SI: Self-incompatibility; SRLP: S-domain receptor-like protein; SRLCK: S-domain receptor-like cytoplasmic kinases; SD: S domain; SLG: S-locus glycoprotein; SCR/SP11: *S* locus cystein rich protein/*S* locus protein 11; KD: Kinase domain; ARC1: Arm Repeat Containing; TM: Transmembrane domain; ARK1/ARK2/ARK3: Arabidopsis Receptor Kinase; MRCA: Most recent common ancestor; A_SRK: Common ancestor of Brassicaceae SRKs; A_SRKI: Ancestor of *SRKI*; A_SRKII: Ancestor of *SRKII*; AtPUB-ARM: Arabidopsis plant U-box/ARM-repeat; RIO1: Right Open Reading 1.

## Competing interest

The authors declare that they have no competing interests.

## Authors’ contributions

SX and PL participated in the design of study, and conducted the phylogenetic analysis. PL and SX prepared the manuscript. ML carried out lab work. All authors participated in reading and approving the manuscript.

## Supplementary Material

Additional file 1: Table S1 List of SRLK and SRLP sequences retrieved from Phytozome v9.0 and homologous RLCK sequences retrieved from Plaza v2.5 databases. Click here for file

Additional file 2: Figure S1 A maximum likelihood (ML) phylogenetic tree of KD sequences from 392 SRLKs and 96 homologous RLCKs. The distribution patterns of SRLKs (filled triangles) and SRLCKs (open triangles) from five plants are shown in the same color scheme as in Figure 2. The domain fusion events are also shown in the same color scheme as in Figure 2. Click here for file

Additional file 3: Figure S2 Inference of gene duplication/loss and fusion events in SRLK evolution. The architectures of leaves are indicated by the minus sign for SRLCKs. Gene fusion events are indicated by filled circles at internal nodes. Gene duplication events are indicated by filled rectangles at internal nodes and gene loss events are indicated by “LOST” at the leaves. Click here for file

Additional file 4: Table S2 List of Brassicaceae SRKs sequences retrieved from UniProt. Click here for file

Additional file 5: Figure S3 A ML S-domain tree constructed by including 7 SRLK members in SD-1 g group and 47 full length SRKs from Brassicaceae species as in Figure 6. The aLRT bootstrap values of the major internal nodes are indicated by numbers. Three main clusters of *Brassica* class-I, *Brassica* class-II, and *Arabidopsis/Capsella* as well as out groups are shown. Click here for file

Additional file 6 **Supporting dataset 1.** Alignment of KD sequences from 392 SRLKs and 96 homologous RLCKs. Click here for file

Additional file 7 **Supporting dataset 2.** Alignment of KD sequences from 47 Brassicaceae SRK sequences. Click here for file
