# Supplementary figures and images for: Evolution of S-domain receptor-like kinases in land plants and origination of S-locus receptor kinases in Brassicaceae
Source: BMC Evol Biol. 2013 Mar 19;13:69. doi: 10.1186/1471-2148-13-69 (PMC3616866; doi:10.1186/1471-2148-13-69)

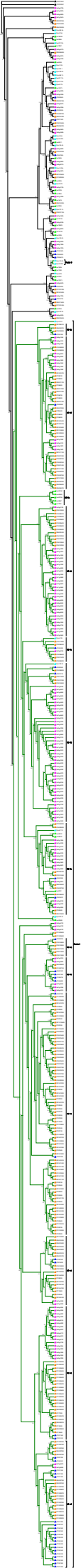

Supplement: Additional file 2: Figure S1 — A maximum likelihood (ML) phylogenetic tree of KD sequences from 392 SRLKs and 96 homologous RLCKs. The distribution patterns of SRLKs (filled triangles) and SRLCKs (open triangles) from five plants are shown in the same color scheme as in Figure 2. The domain fusion events are also shown in the same color scheme as in Figure 2. [file 1471-2148-13-69-S2.pdf]

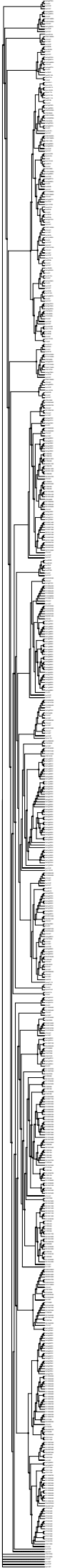

Supplement: Additional file 3: Figure S2 — Inference of gene duplication/loss and fusion events in SRLK evolution. The architectures of leaves are indicated by the minus sign for SRLCKs. Gene fusion events are indicated by filled circles at internal nodes. Gene duplication events are indicated by filled rectangles at internal nodes and gene loss events are indicated by “LOST” at the leaves. [file 1471-2148-13-69-S3.pdf]

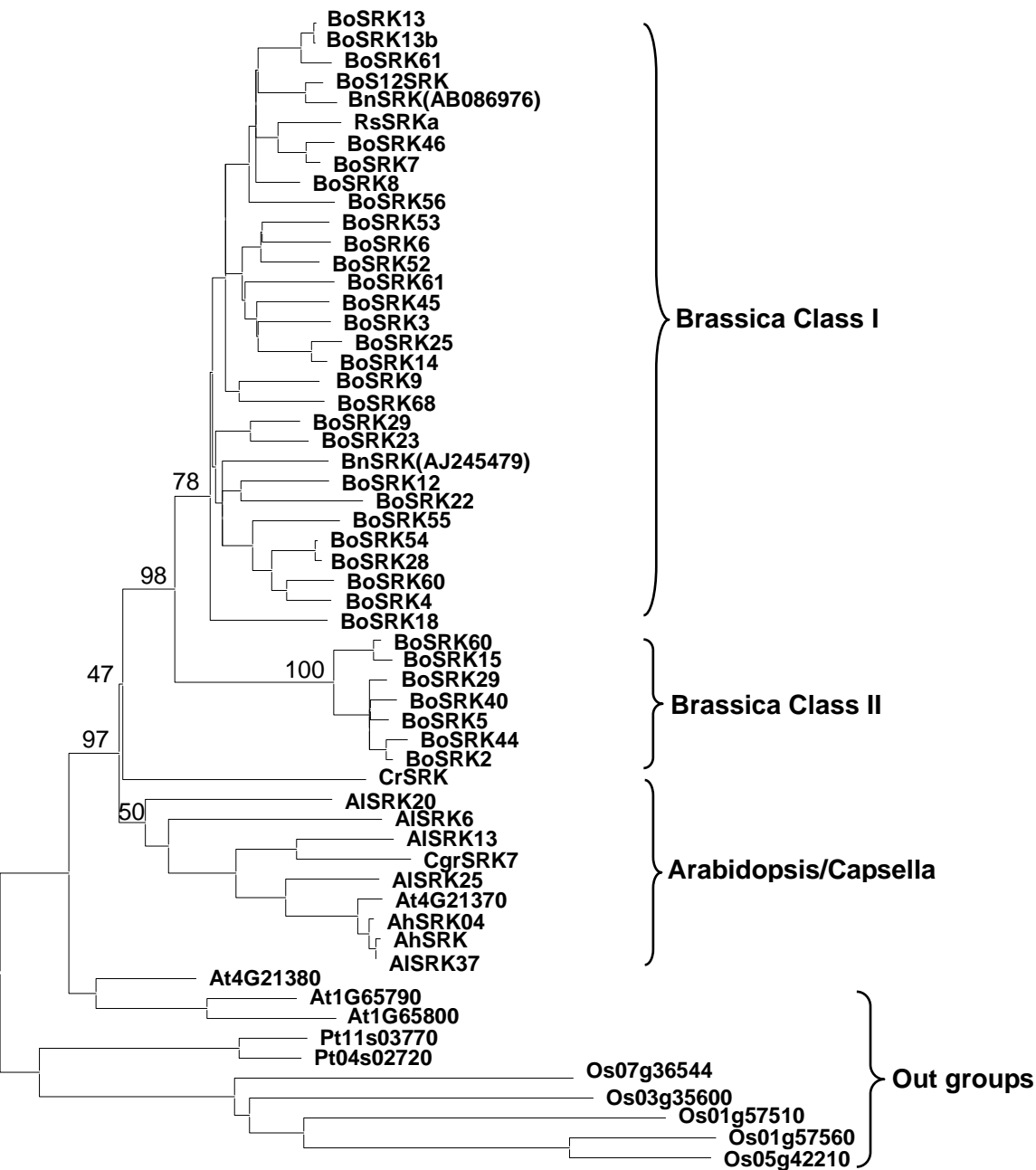

Supplement: Additional file 5: Figure S3 — A ML S-domain tree constructed by including 7 SRLK members in SD-1 g group and 47 full length SRKs from Brassicaceae species as in Figure 6. The aLRT bootstrap values of the major internal nodes are indicated by numbers. Three main clusters of Brassica class-I, Brassica class-II, and Arabidopsis/Capsella as well as out groups are shown. [file 1471-2148-13-69-S5.pdf]
