# Supplementary material for: Evolution of S-domain receptor-like kinases in land plants and origination of S-locus receptor kinases in Brassicaceae
Source: BMC Evol Biol. 2013 Mar 19;13:69. doi: 10.1186/1471-2148-13-69 (PMC3616866; doi:10.1186/1471-2148-13-69)

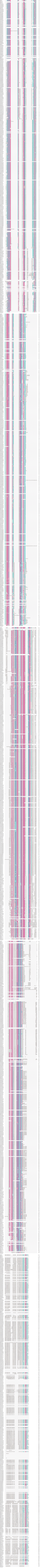

Supplement: Additional file 6 — Supporting dataset 1. Alignment of KD sequences from 392 SRLKs and 96 homologous RLCKs. [file 1471-2148-13-69-S6.pdf]
