# Supplementary material for: Evolution of S-domain receptor-like kinases in land plants and origination of S-locus receptor kinases in Brassicaceae
Source: BMC Evol Biol. 2013 Mar 19;13:69. doi: 10.1186/1471-2148-13-69 (PMC3616866; doi:10.1186/1471-2148-13-69)
Supplement: Additional file 7 — Supporting dataset 2. Alignment of KD sequences from 47 Brassicaceae SRK sequences. [file 1471-2148-13-69-S7.pdf]

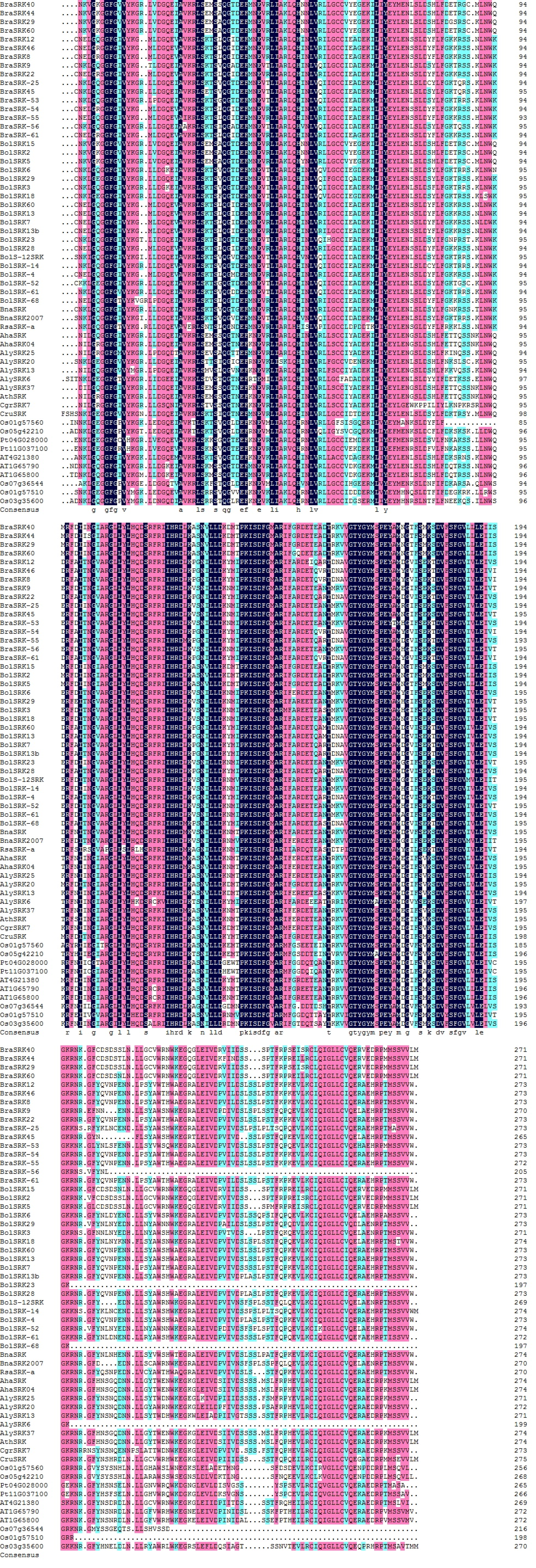
BraSRK40
BraSRK44
BraSRK29
BraSRK60
BraSRK12
BraSRK46
BraSRK8
BraSRK9
BraSRK22
BraSRK-25
BraSRK45
BraSRK-53
BraSRK-54
BraSRK-55
BraSRK-56
BraSRK-61
BolSRK15
BolSRK2
BolSRK5
BolSRK6
BolSRK29
BolSRK3
BolSRK18
BolSRK60
BolSRK13
BolSRK7
BolSRK13b
BolSRK23
BolSRK28
BolS-12SRK
BolSRK-14
BolSRK-4
BolSRK-52
BolSRK-61
BolSRK-68
BnaSRK
BnaSRK2007
RsaSRK-a
AhaSRK
AhaSRK04
AlySRK25
AlySRK20
AlySRK13
AlySRK6
AlySRK37
AthSRK
CgrSRK7
CruSRK
Os01g57560
Os05g42210
Pt04G028000
Pt11G037100
AT4G21380
AT1G65790
AT1G65800
Os07g36544
Os01g57510
Os03g35600
Consensus
